# Performing Through Privatization: An Ecological Natural Experiment of the Impact of the Swedish Free Choice Reform on Ambulatory Care Sensitive Conditions

**DOI:** 10.3389/fpubh.2021.504998

**Published:** 2021-05-31

**Authors:** Paola A. Mosquera, Miguel San Sebastian, Bo Burström, Anna-Karin Hurtig, Per E. Gustafsson

**Affiliations:** ^1^Department of Epidemiology and Global Health, Umeå University, Umeå, Sweden; ^2^Department of Public Health Sciences, Equity and Health Policy Research Group, Karolinska Institutet, Stockholm, Sweden

**Keywords:** health system reform, ambulatory care sensitive conditions, natural experiment, interrupted time series analysis, Sweden

## Abstract

**Background:** In 2010, Sweden opened up for establishment of privately owned primary health care providers, as part of a national Free Choice in Primary Health Care reform. The reform has been highly debated, and evidence on its effects is scarce. The present study therefore sought to evaluate whether the reform have impacted on primary health care service performance.

**Methods:** This ecological register-based study used a natural experimental approach through an interrupted time series design. Data comprised the total adult population of the 21 counties of Sweden 2001–2009 (pre-intervention period) and 2010–2016 (post-intervention period). Hospitalizations and emergency department visits for ambulatory care sensitive conditions (ACSC) were used as indicators of primary health care performance. Segmented regression analysis was used to assess the effects of the reform, in Sweden as a whole, as well as compared between counties grouped by (i) change in private provision pre- to post reform; (ii) the timing of the implementation; and (iii) sustained presence of private providers both pre- and post-reform.

**Results:** The results suggest that, following the introduction of the reform in Sweden as a whole, the trends in total hospitalizations rates were slowed down by 1.0% albeit acute emergency visits increased 1.1% more rapidly after the introduction of the reform. However, we found no evidence of more beneficial effects in counties where the reform had been implemented more ambitiously, specifically those with a larger increase in private primary care providers, or where the reform was introduced early and thus had longer time effects to emerge. Lastly, counties with a sustained high presence of private primary care providers displayed the least favorable development when it comes to ACSC.

**Conclusion:** Taken together, the present study does not support that the Swedish Free Choice reform has improved performance of the primary care delivery system in Sweden, and suggests that high degree of private provision may involve worse performance and higher care burden for specialized health care. Further evaluations of the consequences of the reform are dire needed to provide a comprehensive picture of its intended and unintended impact on health care provision, delivery and results.

## Background

The Swedish health care system has historically been predominantly public, managed and provided by the 21 county councils, but has since the 1990s gradually increased its market-orientation, particularly within primary health care (1). In 2007–2009, a few counties introduced primary care choice models for patients and opened up the market for new establishment of private primary health care providers. On January 1st 2010, Sweden opened up for the establishment of privately owned primary health care (PHC) providers, as part of a national Free Choice in PHC (FCPHC) reform. The reform was implemented nationally in conjunction with a Health Care Guarantee law ensuring the promptness of access to health care [1]. While such notable changes in the health system can be expected to impact on health care access, quality and performance, the national effects of the reform, and particularly the results of the health care, have been poorly studied (1–3).

The FCPHC reform consisted of two main parts, directed at providers and patients, respectively. First, the reform allowed all PHC providers that met certain basic requirements to establish a health care center at a geographical location of their choice; and second, it allowed patients to choose their PHC provider, which in turn served as a basis for capitation payment to the providers (4). The reform thus involved a shift in the responsibility of the PHC centers; from the entire population in a catchment area, to only the patients listed at that specific center. The main motivations underlying the reform were to expand the PHC provision and to introduce competition between health care centers, and thereby improve efficiency and quality of services, which ostensibly would lead to increased access and stronger status for the patients (2, 5). An increased access and efficiency could in turn be expected to involve a shift of burden of care from secondary and tertiary to primary care.

The reform has been a matter of contention even since before its implementation. One reason for this is that the introduction of the reform embodies an underlying ideological shift from a more egalitarian to a more libertarian view of health care organization (5). Other concerns were also raised about reduced continuity, increased fragmentation and impaired equity in the provision of services (4, 6–8). Initial reports by public agencies indicated that implementation of the reform was coupled with increased new establishments of private health care providers, increased number of health care visits, and a maintained level of quality, but with an inconclusive and debated effect on the actual gains in terms of health care performance (3, 4, 8). Moreover, recent studies including those summarized in a scoping review in 2017 suggested that the increased number of PHC visits had been concentrated particularly to areas with a high patient-provider ratio and to socioeconomically advantaged groups (2, 9, 10). When it comes to quality and performance, one early assessment (up to 2013) found small improvements of patient' satisfaction with care but no significant effects on avoidable hospitalizations or satisfaction with access to care (9). Furthermore, it has also been suggested that the reform had negatively affected provision of services for patients with complex needs, which may in turn have lessened the impact of PHC on overall population health (2, 11).

The introduction of similar choice policies and market competition in other European health systems has, like in Sweden, been controversial. The accumulated evidence on the effects is so far insufficient and inconclusive, and has been limited to outcomes related to health service provision such as waiting times and patient satisfaction (4, 12–14). Therefore, knowledge about how PHC service delivery performance is affected by the reform is dearly needed for public health practice and policy-making. Particularly in light of the possible detrimental effects of the reform on equity provision, whether the reform has actually led to improvement in overall PHC performance is key evidence to make a comprehensive assessment of its impact.

While there are many alternative approaches to evaluate policies and interventions, there is a growing interest from epidemiologists and public health researchers in using natural experiments. Natural experiment is an approach referring to empirical studies that evaluate the effects of interventions through detailed comparisons of contrasting cases (e.g., exposed/non-exposed), where the intervention is not allocated by the researcher but naturally occurring (15–19). In the Swedish case, the FCPHC reform permitted and sought to stimulate—but did not enforce—increased establishment of private PHC providers. Its introduction indeed led to large shifts from public to private PHC provision in certain counties, but minimal changes in others. Moreover, a number of counties had already introduced free choice models for patients years before the FCPHC reform implementation, and some of them had had a sustained and continuous high presence of private providers over time. This unintended variation in the de facto implementation of the reform creates a design opportunity to detect and disentangle potential reform effects from underlying secular trends according to a natural experimental approach.

The present study sought to utilize a natural experiment design to evaluate whether the reform have impacted PHC service performance at the national level, and if the impact varied by regional differences in the de facto implementation of the reform. We measured ambulatory care sensitive conditions (ACSC), which are widely used to assess overall access, quality and performance of the primary care delivery system (20, 21). Hospitalizations for ACSCs are commonly divided into acute and chronic ACSCs, which reflect different aspect of PHC (20). While hospitalizations for acute conditions (e.g., ear infection) can reflect suboptimal timeliness of PHC and are potentially preventable by e.g., early diagnosis and prompt treatment, hospitalizations for chronic diseases (e.g., diabetic complications) can relate more to poor effectiveness of PHC, as it relies on continuous monitoring, patient education and control (20). We hypothesize that the reform has led to reduced rates of avoidable hospitalizations and emergency department visits for ACSC (for example through the increase of access and number of visits to primary care). We further hypothesize that the effects may show some regional variations in impact according to different levels of exposure in de facto implementation of the reform (i.e., greater improvements in counties with more marked and enduring implementation).

With these hypotheses as a point of departure, the present study aimed to examine: (1) whether the FCPHC reform has led to national decreased rates of avoidable hospitalizations and emergency visits in Sweden 2001–2016, and (2) whether the effects of the reform shows regional variations depending on (a) increase in public/private ratio (b) timing of implementation of the reform, and (c) sustained presence of private PHC providers.

## Materials and Methods

### Design and Data

This ecological register-based study was based on data on the total adult population of the 21 counties of Sweden aged 20 years and older for the time period 2001–2016. As a majority of young Swedes still go to secondary school and lives with their parents up to the age of 19 years, 20 years was chosen as the youngest age. We employed a natural experimental approach to assess the effects of the FCPHC reform; specifically, an interrupted time series design with segmented regression analysis.

Interrupted time series analysis (ITSA) is the strongest quasi-experimental approach for evaluating longitudinal effect of public health policies introduced at a population level over a clearly defined period of time (16–19). A single-group ITSA estimates an intervention effect by the trends in the outcome over a period of time following the intervention, as compared to the trends before the intervention (19), whereas a multiple-group ITSA additionally compares the trends before and after the intervention between a treated group and one or more control groups (unexposed or less exposed) (19).

In the present study we used single-group ITSA to test the overall national reform effects, and a series of multiple-group ITSA comparing counties with different levels of the de facto implementation of the reform, in order to more specifically attribute any effects to the FCPHC reform.

Aggregated county-level data was collected for each year of observation 2001–2016. Outcome data stratified by sex and 5-year age groups was retrieved from the National Patient Register of the National Board of Health and Welfare. Total population numbers (for denominators) were retrieved from publicly available data from Statistics Sweden; and information on numbers of annual private and public PHC centers per county and hospital beds per 1,000 population were gathered from the Swedish Association of Local Authorities and Regions.

The studies involving human participants were reviewed and approved by The Regional Ethical Review Board in Umeå (approval ref. no. 2017/229-31). Written informed consent for participation was not required for this study in accordance with the national legislation and the institutional requirements.

### Variables

#### Intervention Operationalization

To operationalize the FCPHC reform, contrasting groups of counties were created according to three characteristics of the de facto implementation of the reform (1, 8).

##### Magnitude of Implementation

While the reform covered the entire Sweden from January 1st 2010, the regional context varies when it comes to the attractiveness for private health care providers to establish (1). As a results, the de facto implementation of the reform as expressed in a relative shift from public to private providers remained very unevenly distributed across the country, with some counties experiencing large increase in the proportion of private providers, while in other counties the presence of private providers did not change noticeably.

A number of steps were conducted to operationalize the magnitude, with the purpose of identifying a population for whom the introduction of reform indeed involved a large increase in the exposure to private PHC provision (high magnitude of implementation), and a contrasting population for whom the reform has not involved any major change in this regard (low magnitude of implementation). First, the proportion of private PHC centers was calculated for each county (i.e., Number of private PHC centers/Number of private + Number of public PHC centers). Second, the change in the proportion of PHC from before to after the reform was calculated for each county, using the year before the implementation of the reform (i.e., 2009 for the majority of counties) as the baseline proportion, and the average proportion across the years following the reform (i.e., 2010–2016 for the majority of counties) as the post-reform proportion. As absolute and relative changes may capture different aspects of increased/decreased privatization, both the absolute (before – after) and relative (before/after) change in the proportion of private PHC centers from before to after the reform were calculated separately. Third, the 21 counties were ranked according to the absolute and relative changes, respectively, and then a mean of the two rankings was calculated. Fourth and last, the mean rank was divided into tertiles. The top tertile encompassing the seven counties with greatest increase in the private proportion from before to after the reform (>10% absolute increase and >60% relative increase) was categorized as high magnitude (=1); the lowest tertile of the seven counties with the smallest increase (or even a decrease) in the private proportion (<6% absolute increase and <15% relative increase) was categorized as low magnitude (=0). To ensure a clear contrast for the comparison, the middle tertile consisting of counties with a moderate change in the private proportion was excluded of the comparison (see Table 1; counties by groups based on 2016 figures).

**Table 1:** Counties by groups of intervention exposure.

**County**	**Number of health care providers**		**% Private providers**		**Classification**		
	**Before^a^**	**After^b^**	**Before^a^**	**After^b^**	**Magnitude**	**Time**	Sustained presence before/after^*^
Jönköping	32	50	6.3	37.5	High	Late	Low/Low
Södermanland	21	26	9.5	33.7	High	Late	Low/Low
Dalarna	30	29	0.0	15.3	High	Late	Low/Low
Kronoberg	26	32	11.5	32.1	High	Early	Low/Low
Uppsala	41	42	24.4	48.5	High	Early	Low/High
Västra Götaland	157	202	25.5	43.7	High	Early	Low/High
Värmland	35	38	11.4	25.0	High	Late	Low/Low
Norrbotten	33	33	3.0	12.4	Middle	Late	Low/Low
Skåne	125	150	27.2	42.2	Middle	Early	Low/High
Gävleborg	37	40	18.9	33.2	Middle	Late	Low/Low
Stockholm	171	202	49.1	64.1	Middle	Early	High/High
Västernorrland	26	32	23.1	35.7	Middle	Late	Low/Low
Östergötland	42	43	14.3	20.4	Middle	Early	Low/Low
Västerbotten	36	38	11.1	17.1	Middle	Late	Low/Low
Blekinge	21	21	33.3	39.2	Low	Late	High/Low
Halland	44	47	43.2	48.6	Low	Early	High/High
Västmanland	30	30	53.3	58.3	Low	Early	High/High
Gotland	8	7	25.0	27.2	Low	Late	Low/Low
Jämtland	26	26	15.4	17.0	Low	Late	Low/Low
Örebro	29	29	13.8	13.7	Low	Late	Low/Low
Kalmar	46	39	41.3	31.9	Low	Late	High/Low
Total Sweden	1095	1163	28.5	41.4	N/A	N/A	N/A

a *Year before implementation of the reform*.

b *Average across the years after implementation of the reform*.

* *Higher than Swedish average*.

##### Timing of Implementation

A number of counties introduced a free choice model and opened up the market for new establishment of private health care providers before the national implementation of the FCPHC reform; in 2007 (Halland), 2008 (Stockholm and Västmanland) and 2009 (Uppsala, Kronoberg, Skåne, Östergötland and Västra Götaland) (22). The populations of these eight counties were thus exposed to free choice and private PHC provision before the rest of the Swedish population, and were categorized as early implementers (=1). The other 13 counties, which implemented in 2010 when the reform become national, were categorized as late implementers (=0).

##### Sustained Presence

A third group of counties was identified based on sustained high presence of private PHC providers over the entire observation period. These counties had in effect already exemplified a model prescribed by the reform. They were special in that they were highly exposed to private health care provision both in time and magnitude, but at the same time a saturated market may have diminished opportunities for incremental changes in the establishment of private providers following the reform. Three counties had a private/public proportion above the national mean both before (28.5%) and after (41.4%) the reform, and were therefore categorized as having a sustained presence (=1), while 13 counties with a lower than national average in private/public proportion both before and after the reform were categorized as having a low presence of private providers (=0). Counties that did not have consistently higher or lower proportions than national averages were excluded of the comparison.

#### Outcome

Hospitalizations and emergency department visits for ambulatory care sensitive conditions (ACSC) were used as outcomes. ACSC are a set of acute and chronic health conditions that potentially can be managed with timely and effective primary health care, reducing the need for or avoiding secondary care such as hospitalization and emergency department visits (20, 21). These outcomes were chosen as global and results-oriented indicators of PHC performance as they are widely used to assess access, quality and performance of the primary care delivery system (20, 23).

The identification of ACSC was done through International Classification of Disease (ICD-10) codes and categorized by the Swedish National Board of Health and Welfare classification of avoidable hospitalizations. This classification has been used for monitoring and research on primary care performance and quality in Sweden (24, 25) and is comparable to some other international classifications (26).

Chronic ACSC include diabetes complications, hypertension, heart failure, chronic obstructive pulmonary disease, angina, anemia and asthma. Acute ACSC include diarrhea, bleeding ulcers, epileptic seizures, pelvic inflammatory diseases, pyelonephritis and ear, nose and throat infection (20). Hospitalizations for ACSC (ACSC-H, “avoidable hospitalizations”) were defined as all inpatient stays for any of the conditions listed above. Emergency department visits due to ACSC (ACSC-EV) were defined using as a proxy all ambulatory care visits to hospital that have not been scheduled in advance, and that have as diagnosis any of the conditions listed above. Acute and chronic avoidable hospitalizations and emergency department visits were analyzed separately as the temporal effects of the reform can be expected to differ for these groups of disease.

#### Analysis

Age-standardized hospitalization and emergency visits rates (per 100,000 inhabitants) were calculated across the period 2001–2016, using Swedish total population 2016 as standard.

Corresponding to the first aim, we first estimated the change in trends of ACSC-H and ACSC-EV in Sweden as a whole through single-group ITSA. Corresponding to the second aim we subsequently performed multiple-group ITSA for comparisons between contrasting groups of counties with different levels of the de facto implementation of the reform as follows: (a) High vs. low magnitude of implementation; (b) Early vs. late timing of implementation; and (c) High vs. low sustained presence of private providers. The pre-intervention period was defined as 2001–2009 and post-intervention period 2010–2016. Time series for each outcome (avoidable hospitalizations and emergency visits) were plotted by 6-month periods, giving 18 points before and 14 points after the reform implementation. Analysis for each outcome was done by total number of cases as well as separately by acute and chronic conditions, totaling six single-group and eighteen multiple-group ITSA models.

Preliminary analysis suggested that our data was moderately overdispersed, and we therefore fit our segmented regression analysis with negative binomial regression models, which is an approach that has been shown to be appropriate for modeling time series of counts data when there is evidence of overdispersion (27). The presence of autocorrelation within the data was examined by plotting the residuals and robust standard errors were calculated to control for mild violations of underlying assumptions. Rate ratios (RR) and the 95% confidence intervals (95% CI) were obtained using the Stata 15.0 software.

Since the number of available hospital beds could be a particularly important confounder for hospitalization rates (25), ACSC-H models were adjusted for number of hospital beds per 1,000 inhabitants. Additional confounders that were considered in the analysis were the median income, the percentage of people born outside Sweden and percentage of people who attained highest level of education per county. There were no significant differences among the groups for any of these characteristics so they were excluded from the main analysis.

##### Auxiliary Analyses

In addition to the six single-group ITSA and 18 multiple-group ITSA carried out as the main analyses, a number of additional analyses were run. First, the models were rerun stratified by sex (women and men; in total 48 analyses) and broad age groups (<65 and >65 years; 48 analyses), to explore whether the overall results were valid across gender and age.

Moreover, to explore the sensitivity of the results to the choice of cut-offs for the intervention operationalization and the time point to evaluate the reform, a series of analyses were carried out using alternative cut-offs. For the intervention operationalization, the cut-off for early implementation was changed to at least 1 year before 2010 (i.e., implemented before 2009 rather than before 2010; in total 24 analyses), and for the magnitude of implementation the cut-off was changed to the national average, including all counties (rather than comparing the highest and lowest tertile of counties; 24 analyses). For the time point to evaluate the reform, the year was changed to 2011 instead of 2010, allowing for a longer period of implementation (24 analyses).

Since the auxiliary analyses comprised a large set of models and led to similar conclusions as the main analyses, a summary of the results are reported in the Results section, and details are available on request.

## Results

### Overall Impact of the Reform in Sweden

**Figure 1:**
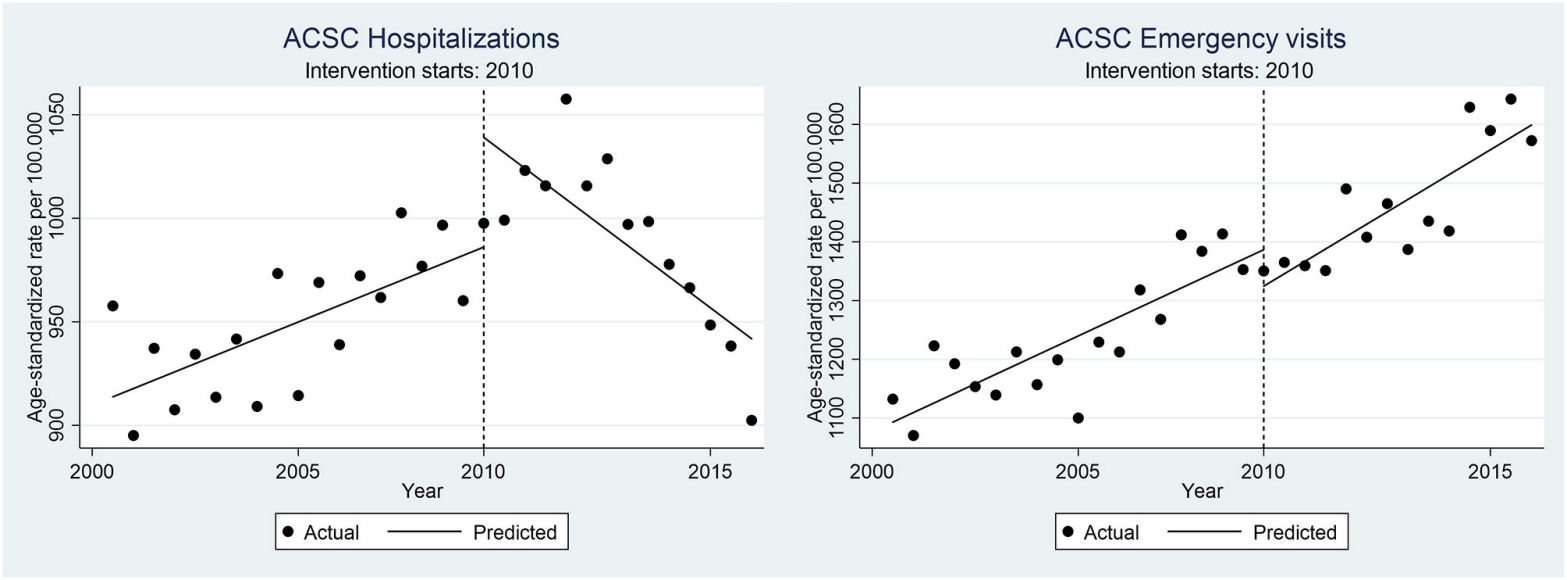
Age-standardized ACSC-H and ACSC-EV rates (per 100,000 population) in Sweden before-after the reform implementation.

A summary of results of the single-group ITSA, corresponding to the first aim of estimating the overall impact of the FCPHC reform on ACSC outcomes in Sweden as a whole, are reported in Table 2 and Figure 1.

**Table 2:** Segmented regression analysis of ACSC-H and ACSC-EV trends in Sweden before and after reform implementation.

**Sweden**	**ACSC hospitalizations^a^**			**ACSC emergency visits**		
	**Total**	**Chronic**	**Acute**	**Total**	**Chronic**	**Acute**
	**RR (95% CI)**	**RR (95% CI)**	**RR (95% CI)**	**RR (95% CI)**	**RR (95% CI)**	**RR (95% CI)**
Pre-trend (2001–2009)	1.009 (1.003, 1.015)	1.004 (0.997, 1.011)	1.022 (1.015, 1.028)	1.013 (1.009, 1.017)	1.011 (1.006, 1.016)	1.017 (1.014, 1.020)
Post trend (2010–2016)	0.999 (0.990, 1.009)	0.987 (0.977, 0.998)	1.025 (1.012, 1.037)	1.014 (1.011, 1.018)	1.004 (0.999, 1.009)	1.029 (1.025, 1.032)
Pre-post trend (2001–2009 vs. 2010–2016)	0.990 (0.985, 0.995)	0.983 (0.977, 0.989)	1.003 (0.997, 1.009)	1.001 (0.996, 1.007)	0.993 (0.986, 1.001)	1.011 (1.007, 1.015)

a *Adjusted for No. of hospital beds per year and county*.

The 9 years prior to the introduction of the reform (the “pre-trend”), saw a universal increase in ACSC outcomes, both for hospitalizations and emergency visits, and for chronic as well as acute ACSC. These trends were significant for all outcomes except for chronic ACSC hospitalization, and the largest increases were seen for acute ACSC hospitalizations and emergency visits, corresponding to an estimated 2.2 and 1.7% average semi-annual increase in the age-adjusted rates, respectively.

Upon the introduction of the reform in 2010, the trends changed for the different outcomes. The trend of total ACSC hospitalization flattened out after introducing the reform (2011–2016), as seen in insubstantial and non-significant 0.1% decrease (post-trend). This shift in trend from before to after the introduction of the reform was significant (pre-post trend), estimated at an average 1% lower increase in the semi-annual hospitalization rate, relative to the pre-intervention trend. This effect seemed to be driven by post-reform reductions in hospitalizations due to chronic, rather than acute, ACSC.

In contrast to hospitalization rates, the increasing trend in total ACSC emergency visits before the reform continued even after the introduction of the reform, at a comparable pace and with no significant break in the trend (post-trend). A similar pattern was seen specifically for chronic ACSC emergency visits. In contrast, the increasing trend of acute ACSC emergency visits rather accelerated after the reform, estimated at a significant 1.1% higher increase after compared to before the reform (pre-post trend).

Auxiliary analyses (results not shown; available on request) by age specific groups and stratified by sex, overall pointed in the same direction as in the total population. For example, there was a significant decrease pre-post trend in total ACSC hospitalizations (1.1% in women, 0.9% in men, 1.2% in >65 years and 0.6% in <65 years; compared to 1.0% in the total sample) and in chronic ACSC hospitalizations (1.6% in women, 1.7% in men, 1.8% in >65 and 1.3 in <65; compared to 1.7% in total population), and a significant increase in acute emergency visits (0.7% in women, 1.4% in men, 1% in >65 and 1.2% in <65; compared to 1.1% in the total population). In addition, pre-post trends were also significant for acute hospitalizations in men (0.8% increase; compared to non-significant increase of 0.3% in the total sample) and chronic emergency visits in men and <65 years (0.7 and 1% decrease compared to non-significant decrease of 0.7% in the total population).

### Variation in Impact by Reform Implementation Characteristics

Based on the overall impact of the reform on ACSC outcomes in Sweden described above, the subsequent series of analyses report variations in the impact across counties who differed with respect to increases in the proportion of private PHC providers following the introduction of the reform (magnitude; aim 2a), the year of introduction of the reform (timing; aim 2b), and a long-term presence of private providers (sustained presence; aim 2c).

#### Magnitude of Implementation

**Figure 2:**
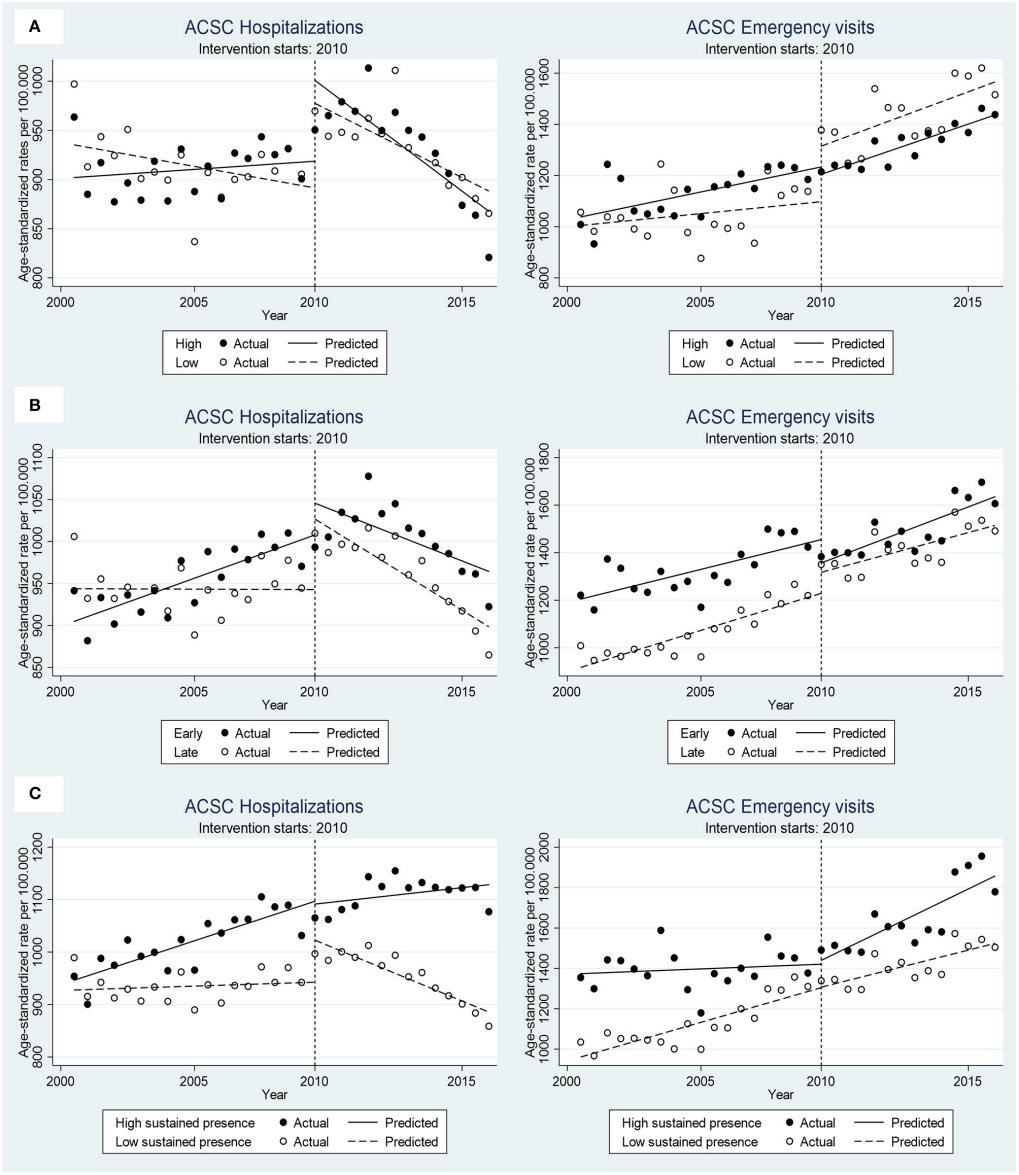
Age-standardized ACSC-H and ACSC-EV rates (per 100,000 population) before-after the reform implementation by comparison groups. **(A)** High vs. low implementers, **(B)** Early vs. late implementers and **(C)** high vs. low sustained presence of private providers.

Table 3 reports the results from multiple-groups ITSA, with magnitude of implementation of the reform as the between-groups contrast. The trends were similar in the two comparison groups for all outcomes, both before (pre-trend difference) and after (post-trend difference) the introduction of the reform in 2010 (Figure 2). This similarity in trends resulted in a small (0.4–0.7%) and non-significant pre-post trend difference, indicating no variation in impact of the reform between the group with larger compared to smaller increase in the proportion of private PHC providers following the introduction of the reform.

**Table 3:** Segmented regression analysis of ACSC-H and ACSC-EV trends comparing counties by magnitude of implementation 2001–2016.

**High vs. low implementers**	**Hospitalizationsy^a^**			**Emergency visits**		
	**Total**	**Chronic**	**Acute**	**Total**	**Chronic**	**Acute**
	**RR (95% CI)**	**RR (95% CI)**	**RR (95% CI)**	**RR (95% CI)**	**RR (95% CI)**	**RR (95% CI)**
Initial mean level difference	0.961 (0.917, 1.006)	0.953 (0.897, 1.013)	0.977 (0.938, 1.017)	1.034 (0.914, 1.170)	0.987 (0.865, 1.127)	1.133 (1.007, 1.273)
Pre-trend high implementation	1.005 (1.001, 1.009)	1.001 (0.996, 1.006)	1.014 (1.010, 1.019)	1.010 (1.003, 1.016)	1.004 (0.996, 1.012)	1.020 (1.014, 1.026)
Pre-trend low implementation	1.001 (0.997, 1.005)	0.999 (0.994, 1.004)	1.007 (1.004, 1.011)	1.005 (0.996, 1.013)	0.997 (0.988, 1.005)	1.020 (1.011, 1.029)
Pre-trend difference	1.003 (0.999, 1.008)	1.004 (0.998, 1.010)	1.001 (0.997, 1.005)	1.005 (0.994, 1.016)	1.008 (0.996, 1.019)	0.999 (0.088, 1.010)
Post-trend high implementation	0.998 (0.993, 1.002)	0.992 (0.988, 0.997)	1.009 (1.000, 1.018)	1.014 (1.011, 1.016)	1.005 (1.001, 1.009)	1.026 (1.024, 1.028)
Post-trend low implementation	0.998 (0.993, 1.003)	0.991 (0.985, 0.997)	1.012 (1.006, 1.018)	1.013 (0.999, 1.028)	1.000 (0.983, 1.017)	1.033 (1.020, 1.045)
Post-trend difference	1.000 (0.993, 1.006)	1.001 (0.995, 1.008)	0.997 (0.987, 1.007)	1.000 (0.986, 1.015)	1.005 (0.987, 1.022)	0.993 (0.981, 1.006)
Pre-post trend difference	0.996 (0.989, 1.004)	0.997 (0.988, 1.006)	0.996 (0.984, 1.006)	0.995 (0.977, 1.014)	0.997 (0.976, 1.018)	0.994 (0.978, 1.011)

a *Adjusted for No. of hospital beds per year and county*.

#### Early Introduction of the Reform

The comparisons between counties which introduced the free choice earlier than the FCPHC reform was implemented nationally displayed a more complex pattern depending on the outcomes; see Table 4 and Figure 2.

**Table 4:** Segmented regression analysis of ACSC-H and ACSC-EV trends comparing counties by timing of implementation 2001–2016.

**Early vs. late implementers**	**Hospitalizations^a^**			**Emergency visits**		
	**Total**	**Chronic**	**Acute**	**Total**	**Chronic**	**Acute**
	**RR (95% CI)**	**RR (95% CI)**	**RR (95% CI)**	**RR (95% CI)**	**RR (95% CI)**	**RR (95% CI)**
Initial mean level difference	0.979 (0.946, 1.013)	0.996 (0.951, 1.044)	0.935 (0.909, 0.962)	1.306 (1.208, 1.411)	1.177 (1.080, 1.283)	1.573 (1.454, 1.702)
Pre-trend early implementation	1.009 (1.003, 1.015)	1.004 (0.996, 1.011)	1.022 (1.013, 1.030)	1.010 (1.006, 1.015)	1.009 (1.003, 1.015)	1.012 (1.008, 1.016)
Pre-trend late implementation	1.003 (1.001, 1.006)	1.001 (0.997, 1.004)	1.009 (1.007, 1.012)	1.016 (1.010, 1.021)	1.010 (1.004, 1.016)	1.027 (1.021, 1.034)
Pre-trend difference	1.006 (1.002, 1.009)	1.006 (1.002, 1.011)	1.005 (1.001, 1.008)	0.994 (0.987, 1.002)	0.999 (0.991, 1.007)	0.985 (0.978, 0.992)
Post-trend early implementation	0.998 (0.994, 1.002)	0.990 (0.986, 0.995)	1.014 (1.008, 1.021)	1.014 (1.010, 1.018)	1.004 (0.999. 1.010)	1.028 (1.024, 1.031)
Post-trend late implementation	0.993 (0.990, 0.997)	0.985 (0.980, 0.990)	1.009 (1.005, 1.013)	1.011 (1.002, 1.020)	0.998 (0.988, 1.008)	1.029 (1.021, 1.037)
Post-trend difference	1.005 (0.999, 1.010)	1.005 (0.999, 1.011)	1.005 (0.998, 1.013)	1.004 (0.994, 1.013)	1.006 (0.995, 1.018)	0.999 (0.991, 1.007)
Pre-post trend difference	0.999 (0.993, 1.006)	0.999 (0.991, 1.007)	1.001 (0.992, 1.009)	1.009 (0.997, 1.021)	1.007 (0.993, 1.021)	1.014 (1.003, 1.025)

a *Adjusted for No. of hospital beds per year and county*.

For all ACSC hospitalization outcomes (total, chronic, and acute), the early adopters of the reform showed a less promising development, with a significantly worse trends before the reform (pre-trend difference). The universal introduction of the reform in 2010 did however not change this pattern, with similarly sized but non-significantly worse trends among the early compared to late implementers (post-trend difference), and a non-significant pre-post trend difference as a result.

In contrast, the early implementers displayed a less pronounced increase in the acute ACSC emergency visits compared to the late implementers before the reform (pre-trend difference), but a similar increase after the reform (post-trend difference). This resulted in a significantly more unfavorable impact of the reform among the early implementers (pre-post trend difference), amounting to a 1.4% accelerated rates among early compared to late implementers. This overall pattern was in the same direction but non-significant for total and chronic ACSC emergency visits.

#### Sustained Presence of Private Providers

The impact of the reform on ACSC outcomes differed consistently between counties with a high-sustained presence of private providers compared to those with a consistently low presence, both before and after the introduction of the reform; see Table 5 and Figure 2.

**Table 5:** Segmented regression analysis of ACSC-H and ACSC-EV trends comparing counties by sustained presence of private providers.

**High vs. low sustained presence o f private providers**	**Hospitalizations^a^**			**Emergency visits**		
	**Total**	**Chronic**	**Acute**	**Total**	**Chronic**	**Acute**
	**RR (95% CI)**	**RR (95% CI)**	**RR (95% CI)**	**RR (95% CI)**	**RR (95% CI)**	**RR (95% CI)**
Initial mean level difference	1.081 (1.042, 1.120)	1.088 (1.039, 1.139)	1.062 (1.021, 1.105)	1.413 (1.315, 1.517)	1.232 (1.136, 1.336)	1.769 (1.631, 1.919)
Pre-trend H/H implementation	1.010 (1.005, 1.015)	1.006 (>1.000, 1.013)	1.018 (1.013, 1.023)	1.002 (0.998, 1.006)	1.002 (0.997, 1.007)	1.001 (0.997, 1.006)
Pre-trend L/L implementation	1.005 (1.003, 1.007)	1.003 (>1.000, 1.005)	1.009 (1.007, 1.012)	1.017 (1.011, 1.022)	1.012 (1.006, 1.018)	1.025 (1.019, 1.031)
Pre-trend difference	1.006 (1.002, 1.010)	1.008 (1.003, 1.012)	1.003 (0.999, 1.006)	0.985 (0.979, 0.992)	0.990 (0.983, 0.998)	0.977 (0.969, 0.984)
Post-trend H/H implementation	1.008 (1.005, 1.011)	1.002 (0.998, 1.006)	1.021 (1.017, 1.024)	1.019 (1.013, 1.026)	1.012 (1.003, 1.020)	1.030 (1.025, 1.035)
Post-trend L/L implementation	0.994 (0.991, 0.997)	0.986 (0.981, 0.990)	1.009 (1.006, 1.013)	1.012 (1.005, 1.018)	1.000 (0.992, 1.008)	1.029 (1.023, 1.035)
Post-trend difference	1.014 (1.010, 1.019)	1.017 (1.011, 1.023)	1.011 (1.007, 1.016)	1.008 (0.998, 1.016)	1.012 (1.001, 1.024)	1.001 (0.993, 1.008)
Pre-post trend difference	1.008 (1.002, 1.014)	1.009 (1.001, 1.017)	1.009 (1.003, 1.015)	1.022 (1.011, 1.034)	1.022 (1.008, 1.036)	1.024 (1.014, 1.035)

a *Adjusted for No. of hospital beds per year and county*.

For hospitalizations outcomes, this was explained by significantly more steeply increasing pre-reform trends among the counties with high compared to low sustained presence of private providers (pre-trend difference), in combination with increasing trends after the reform (post-trend difference), which resulted in a significant 0.8–0.9% less favorable impact of the reform among the counties with sustained presence (pre-post trend difference).

A more sizable less favorable impact of the reform among the counties with high compared to low sustained presence of private providers was also seen for emergency visit outcomes amounting to a 2.2–2.4% difference (pre-post-trend difference). This was the result of less pronounced increasing trends before the implementation (pre-trend difference) in combination with steeper increasing trends after the reform in the counties with sustained presence of private providers (post-trend difference).

#### Sensitivity Analyses

Analyses stratified by sex and age (results available on request) led to the same overall conclusions as in the total population. Specifically, there were no variation in impact of the reform by magnitude of implementation either by gender or age; an unfavorable impact of the reform in acute emergency visits among the early compared to late implementers (1.5% in women, 1.3% in men, 1.% in <65 and 1.1% in >65; compared with 1.4% in the total population); and an overall negative impact of the reform in both, hospitalizations (0.8–0.9% in women and 0.8–1% in men; 1–1.1% in >65; compared with 0.8–0.9 in total population) and emergency visits (2.1–2.5% in women and 2.3–2.4% in men, 2.6–2.7 in <65 and 1.8–1.9% in >65; compared with 2.2–2.4% in total population) among the counties with high compared to low sustained presence of private providers.

The series of sensitivity analysis (results available on request) using different cut-off points for intervention operationalization and timing of the intervention confirmed the inferences from the main analysis, only with slightly larger pre-post trend differences observed for certain comparisons when the time point to evaluate the reform was changed to 2011 instead of 2010, thereby allowing for a longer period of implementation. Specifically, the pre-post trend differences were more pronounced for hospitalizations in the high compared to low sustained presence counties (1.1–1.3% increase; compared to 0.8–0.9%) and for emergency visits in the early compared to late implementers (1.2–1.5% increase; compared to 0.9–1.4%).

## Discussion

### Summary of Main Findings

This study set out to evaluate the impact of the 2010 Swedish Free Choice in Primary Health Care reform on PHC performance as indicated by rates of hospital admissions and emergency visits for chronic and acute Ambulatory Care Sensitive Conditions. The results suggest that, following the introduction of the reform in Sweden as a whole, the trends in total hospitalizations rates were slowed down and for chronic conditions even turned to a downward trend, which marks a break from the pre-reform secular trend. On the other hand, acute emergency visits increased more rapidly after the introduction of the reform. However, we found no evidence of more beneficial effects in counties where the reform had been implemented more ambitiously, specifically those with a larger increase in private primary care providers, or where the reform was introduced early and thus had longer time effects to emerge. This suggests that the overall changes in Sweden as a whole are not to be attributed to the reform itself. Lastly, counties with a sustained high presence of private primary care providers displayed the least favorable development when it comes to ACSC. Taken together, our study does not provide evidence that the FCPHC reform has had the expected positive impact on ACSC, and instead tentatively suggests that widespread private PHC provision could, in the long run, negatively affect PHC performance.

### Overall National Impact of the Reform

The pattern of slightly increasing trends in hospitalizations for chronic ACSC turning toward downward trend after 2010 have been noted before (28), and has been interpreted as reflecting improved interventions in outpatient care for diseases such as chronic heart failure and chronic obstructive lung disease. The fact that we were unable to attribute this change or the increase in emergency visits to features of the reform overall corresponds to findings from a preliminary study following avoidable hospitalizations up to 2013 (9), and warrants a comment on other possible causes of the observed changes. First, as noted above, the positive development seen for to chronic conditions hospitalizations could reflect universal improvements in the treatment and management of chronic conditions not restricted to primary care. Second, despite a Healthcare Guarantee Law serving to reduce waiting times in health care was introduced in 2010, waiting times in primary and secondary care have rather increased in the years after 2010 (29), a development that possibly could contribute to the increase in emergency care visits. Third, the reduced availability of hospital beds could be expected to impact the tendency to hospitalize patients (25); however, the results remained after adjustment for hospital beds, and this is thus an unlikely contributor to the findings.

Complementary interventions implemented together or as a consequence of the reform could have also played a role on the observed results. For example, it is possible that the reduction of hospitalizations would be a reflection of guideline adaptations made at county-level to improve quality indicators. i.e., the ACSC have been used as quality performance indicators (25) and therefore it is possible that hospital admission criteria for these conditions may have become stricter. Such a behavior could partly explain the combination of decreased chronic hospitalization but increased acute hospitalizations and emergency visits. Moreover, the overall trend of increased emergency visits also could be understood from the fact that stricter admission criteria would result in most of the acute ACSC situations being managed in the emergency rooms, with only very critical cases ending up in hospitalization. Identifying whether such interventions implemented at county-level actually have played a role would require a more deep exploration, e.g., through case studies, since counties have certain degree of autonomy to implement reforms.

### Regional Differences in the Impact of the Reform

When it comes to the between-county comparisons, it is possible that other characteristics than changes in the proportion of private provision have led to the estimated effects of the reform. For example, whereas most of the county councils have a mixed reimbursement system largely based on capitation and a small part on fee-for-service (30), each county decide on their particular arrangements and adapt their system according to their own dynamics, with no national documentation of the various modifications. This makes it difficult to track and operationalize changes in reimbursement systems over time to measure their link to the effects of the reform. Nevertheless, it is well-known that funding mechanisms and reimbursement systems have the potential to create incentives to improve access and quality of care (31). Indeed, recent studies in southern Swedish counties, including Stockholm, whose reimbursement system has been unique up to 2015 [large fraction based on fee-for-service (60%) and the remaining (40%) unweighted capitation], found increases in the number of visits to health care centers associated with changes in the reimbursement system (7, 32). However, the changes did not particularly benefit those with greater health care needs (7). It is possible that changes in reimbursement system might have ended up incentivising short visits among otherwise healthy people, and therefore the group with chronic conditions may have been de-prioritized in primary care, ending up in emergency care instead. Further research exploring regional differences and changes in reimbursement systems, and their impact on ACSC would be needed to provide more insight into this issue.

The observation that counties with sustained presence of private providers showed the worst development when it comes to ACSC was a notable and surprising finding. This comparison does not directly reflect the impact of the 2010 national reform, but should instead be seen as an illustration of the possible long-term outcome of the reform in Sweden as a whole, if all counties were to develop a persistent dominance of private primary care providers. A lot of controversy surrounds the role of the private (for-profit) sector in health services delivery. Indeed previous research have cautioned policymakers of possible negative effects, such as the potential of the private sector to strip away the workforce from the public sector, and their tendency to focus on advantaged groups and over treat patients to generate more profits (33). Some evidence from low- and middle income countries indeed suggests that increasing private health care provision might not meet the promises of ending up in more efficient, accountable or medically effective provision (33, 34). In Sweden, the introduction of similar market-oriented reforms for other social services have also shown to be detrimental for care-takers (30). However, when it comes to health care in Europe, there is still an ongoing discussion and an open call for sound empirical analysis providing answers as to the rather silent question on the long-term consequences of privatization on health care performance (35). Therefore, robust evaluation and continuous learning and development are needed to ensure the current trend of public-private mix of health provision can fulfill its promise of a quality driven health care service model.

Whereas impact on equity in PHC performance was not part of the scope of the present report, it is important to note that failures to meet the needs of underserved population groups, as has been found in previous research in Sweden (2), could hamper a population-wide improvement in performance (11), and inequity in performance could thus be contributing to the lack of positive overall effect of the reform seen in our results. Considering the long-standing concerns about how the reform may negatively impact on equity, future evaluations are specifically needed to shed light on this issue.

### Methodological Considerations

The main strengths of the present study are the longitudinal design spanning over 16 years of follow up; the use of a comprehensive set of outcomes retrieved from Swedish total population registers of good quality; and the interrupted time series designs, which is considered the best available mean of assessing an intervention impact (15, 36).

Some potential limitations should be considered when interpreting our results. First, the analyses were done using population-level rates and can therefore not be used to make individual-level inferences (17). Furthermore, unplanned hospital visits were used as proxy for emergency visits which may have led to an over or under estimation of the rates in these particular outcomes. While case ascertainment can be a matter of concern, a quality control of the Swedish registers is performed routinely to ensure accuracy and completeness (37). Nevertheless, the extent of measurement bias in this outcome is ultimately unknown.

Although the single-group ITSA does not require to have a comparison group to obtain association between an intervention and outcome (19, 38), it is well-known that having a comparison group to serve as the counterfactual is a superior approach to ascertain intervention effects (39). In that sense, further evaluations comparing Sweden with a truly unexposed group, e.g., using a “synthetic international comparator” (40), would be needed to better understand the effects of the FCPHC reform. Nevertheless, identifying truly comparable contexts for external control groups outside of Sweden will involve challenges by itself. It should also be noted that for some outcomes, control groups in the multiple-groups ITSA were not completely comparable to the treatment group when it comes to observed pre-intervention levels and trends, which could raise concerns about the ability of the analysis to draw causal inferences about the relationship between the intervention and the outcomes (19).

Moreover, it is possible that the lack of effect in counties where the reform had been implemented more ambitiously is due to the chosen operationalization of the reform, and that the results therefore also may conceal differential developments in specific sub-groups (e.g., persons with greater health care needs). However, in this paper, we tried various analyses stratified by sex and age subgroups with different cut-off points for magnitude and timing, yet none of them led to a different result. Nonetheless, it should be noted that using other aspects rather than the public/private proportions to operationalize the reform and other specific subgroups for the analysis could possibly yield different inferences.

It is also possible that the reform needs a longer time to produce an impact on PHC performance, perhaps beyond the period of this study. For example, in the shorter term, the reform would be expected primarily to increase the number of providers; while in the medium term, the benefit might be in access to primary care and only in the long term, a clearer benefit in reducing the burden of secondary and tertiary care would be seen. In this analysis, we included 18 observations points (9 years) before and 14 points (7 years) after the reform implementation, which would be considered sufficient to statistically evaluate changes (17). Nevertheless, the need of more time to observe effects in a long-term outcome cannot be ruled out, and we anticipate future research to explore the more long-term effects of the reform.

Lastly, it is difficult to isolate the independent impact of the reform in a dynamic ever-changing health system and society. In fact, to identify competing or complementary interventions to the reform that could be responsible for shifting the time series of the evaluated outcomes is challenging by itself, as there could be national preventive efforts and particular county level interventions, as well variations in reimbursement systems as discussed above, that could be expected to have a similar effect as the reform itself. Relatedly, considering the long study period over 16 years, a change in unconsidered population or health system characteristics could also potentially confound the analyses, as long as the change coincides with the introduction of the reform. Even though the ITS design is based on both within- and between-group comparisons of trends, and the threat to internal validity is considerably lower than for a weaker study design, confounding cannot be ruled out. In preliminary analyses, sociodemographic characteristics did not differ between the comparison groups, but other unobserved potential confounders include for example regional health system differences, such as variations in clinical practice, and also healthcare seeking behavior. Another potential confounder is the actual prevalence and incidence of chronic disease. There are unfortunately no national register data on disease prevalence in Sweden outside the patient registers that were used as outcomes in the study.

These considerations illustrate the inherent challenges of performing population-wide evaluations in real-world settings, which are relevant for future research on the impact of health system changes on PHC performance. The choice of evaluation design is a key methodological issue that may be restricted by the availability of appropriate data. Controlled designs are preferred but formulation of the comparison population requires careful consideration, which poses a challenge also for regionally implemented interventions [see e.g., (41)]. The issue of competing interventions and confounding is an ever-present threat particularly for weaker evaluation designs, e.g., in the absence of control group or with single pre-test and post-test observations rather than trends. Additional methodological considerations include the choice of outcome, e.g., summary indicators such as ACSC used in the present study, or indicators of more specific aspects of PHC performance (21), as well as the quality and coverage of outcome data; the expected temporality of intervention impact of the chosen outcome and follow-up time – e.g., immediate or delayed; and the possibilities of ecological and individual-level analysis. For future evaluations it would be particularly interesting to consider the possibility to conduct an ITSA with multiple treatment analysis or other design alternatives (19).

### Conclusion

The present study contributes to evidence on the effects of a major health care reform, driven by libertarian goals rather than the egalitarian principles that traditionally have been central to Swedish health care system.

Taken together, the present study does not support that the Swedish Choice in PHC reform has improved the overall quality and performance of the primary care delivery system in Sweden, and suggests that high degree of private provision in PHC may lead to worse PHC performance and higher care burden for specialized health care.

The results illustrate the value of using population-level approaches and counterfactual evaluation designs to assess interventions through attributable impact. While evidence-based policy-making free from ideological or ethical guiding principles may neither be realistic nor desirable, rigorous evidence as provided by the present study represents an important aspect for assessing, tailoring and designing effective health policy. Further evaluations of the consequences of the reform are direly needed to provide a comprehensive picture of its intended and unintended impact on health care provision, delivery and results.

## Data Availability Statement

The raw data supporting the conclusions of this article will be made available by the authors upon request, without undue reservation.

## Ethics Statement

The studies involving human participants were reviewed and approved by The Regional Ethical Review Board in Umeå (approval ref. no. 2017/229-31). The study is based on aggregated data on a large scale, and thus does not process any personal data. The Regional Ethical Review Board in Umeå waived the requirement for written informed consent for participants in this study in accordance with Swedish national regulations (Ethical Review Act 2003:460), as the study only retrieved and utilized secondary register data.

## Author Contributions

PM, PG, and MS conceived the study. PM conducted the data analysis, interpretation of the data and drafted the manuscript, with support from PG. PG and MS contributed to the analysis and interpretation of the data and revised the manuscript. BB and AKH participated in the interpretation of the data and revised the manuscript. All authors approved the final draft.

## Conflict of Interest

The authors declare that the research was conducted in the absence of any commercial or financial relationships that could be construed as a potential conflict of interest.

## Abbreviations

ACSC: Ambulatory Care Sensitive Conditions
ACSC-H: Ambulatory Care Sensitive Conditions-Hospitalizations
ACSC-EV: Ambulatory Care Sensitive Conditions-Emergency Visits
FCPHC: Free Choice in Primary Health Care
ICD: International Classification of Disease
ITSA: Interrupted Time Series Analysis
PHC: Primary Health Care.
